# Supplementary material for: CoIn dual-atom catalyst for hydrogen peroxide production via oxygen reduction reaction in acid
Source: Nat Commun. 2023 Aug 8;14:4766. doi: 10.1038/s41467-023-40467-8 (PMC10409757; doi:10.1038/s41467-023-40467-8)
Supplement: Supplementary file 1 — Supplementary Information [file 41467_2023_40467_MOESM1_ESM.pdf]

CoIn Dual-atom Catalyst for Hydrogen Peroxide Production via Oxygen Reduction Reaction in Acid

Jiannan Du[1, #], Guokang Han[1, #, *], Wei Zhang[1], Lingfeng Li[1], Yuqi Yan[1], Yaoxuan Shi[1], Xue Zhang[2], Lin Geng[3], Zhijiang Wang[1], Yueping Xiong[1], Geping Yin[1], Chunyu Du[1, *]

[1] School of Chemistry and Chemical Engineering, Harbin Institute of Technology, Harbin 150001, P.R. China

[2] Center for Materials and Interfaces, Shenzhen Institutes of Advanced Technology, Chinese Academy of Sciences, Shenzhen 518055, P.R. China

[3] School of Materials Science and Engineering, Harbin Institute of Technology, Harbin 150001, P.R. China

[#]These authors contributed equally to this work.

[*]Corresponding authors: Guokang Han (gkhan@hit.edu.cn), Chunyu Du (cydu@hit.edu.cn)

Figure S1 Optimized structure of the single atom models (s-Co, s-CoVac), and the dual-atom models (d-CoAl, d-CoGa, d-CoIn)

Figure S2 Optimized structure of OOH-, OH- and O-adsorbed dual-atom models (d-CoAl, d-CoGa, d-CoIn)

Figure S3 Adsorption energy of OH (ΔG_{OH}) on the three d-CoMp models as a function of potential

Figure S4 Optimized structure of OOH-adsorbed dual-atom models blocked by OH (d-CoAlOH, d-CoGaOH, d-CoInOH)

Figure S5 Charge density difference diagrams of d-CoMpOH demonstrating the change of charge density upon introduction of MpOH moieties. The isosurface are set to be 0.003 e $bohr^{-3}$, where blue and yellow area represent charge depletion and accumulation, respectively.

Figure S6 Synthesis process of CoIn-N-C

Figure S7 SEM image of the CoIn-ZIF8 precursor

Figure S8 Structural characterization of CoIn-N-C (a) X-ray diffraction pattern, (b) N_2 adsorption−desorption isotherms of CoIn-N-C

Figure S9 Colored raster graphic of other select regions in Figure 2d. (a) (c) Z-contrast difference of the Co-In atomic pairs from CoIn-N-C. (b) (d) corresponding intensity profile Note that a.u. represents arbitrary units.

Figure S10 Co and In K-edge XANES analysts. (a) 1st derivatives of Co K-edge XANES for Co, CoO, CoPc, CoIn-N-C and (b) 1st derivatives of In K-edge XANES spectra for In, In_2O_3 and CoIn-N-C

Figure S11 XANES and XPS spectras for the Co-NC. (a) Co K-edge XANES and (b) Co 2*p* XPS spectra of Co-N-C and CoIn-N-C. Note that a.u. represents arbitrary units.

Figure S12 Wavelet transform (WT) contour plots of Co K edge EXAFS of (a) Co, (b) CoO, and In K edge EXAFS of (c) In, (d) In_2O_3

Figure S13 Photograph of in situ flow cell with optics window and electrochemical cell set-up. (a) three-electrode in situ Raman flow cell and (b) three-electrode electrochemical cell in working conditions

Figure S14 In situ EC-SHINERS results of CoIn-N-C Co-NC and In-NC at different condition. Raman spectra of (a) CoIn-N-C, (c) In-N-C and (d) Co-N-C recorded in ambient air (ex-situ) and Ar saturated 0.1 M $HClO_4$ aqueous solution and (b) CoIn-N-C in D_2O solution at different applied potential. Note that a.u. represents arbitrary units.

Figure S15 Electrochemical characterization of CoIn-N-C.(a) Polarizing curves recorded on CoIn-N-C coated disk electrode during potential scan and current response on the Pt ring electrode with a constant potential of 0.6 V in a mixture solution of 0.1 mol L^{-1} KCl and 10 mmol L^{-1} $K_3Fe(CN)_6$ at 1600 rpm, and (b) the experimentally determined collection efficiency (*N*), (c) ORR polarization curves of CoIn-N-C at different rotating rates in O_2-satureted 0.1 mol L^{-1} $HClO_4$, and (d) the corresponding K-L plots for the calculation of

electron transfer number (n)

Figure S16 Physical characterization of CoIn-N-C Co-N-C and In-NC. (a) XPS survey spectra of Co-N-C, CoIn-N-C and In-NC (b) N 1*s* XPS spectra and fitting results. (c) N content ratio, (d) Raman spectra of Co-N-C, In-NC and CoIn-NC. (e) N_2 adsorption/desorption isotherms and (f) pore distribution curves. Note that a.u. represents arbitrary units.

Figure S17 DFT calculations. (a) Dual atomic models with short (CoIn), middle (CoIn-m) and far (CoIn-f) distances and (b) calculated volcano plot and (c) free energy diagram.

Figure S18 H_2O_2 production performance of CoIn-N-C before and after the accelerated degradation tests (ADTs)

Figure S19 HAADF-STEM images and colored raster graphic of Co-In pair of catalysts after ADTS. The scale bars in the enlarge images (marked areas 1 2 3) are 0.35 nm.

Figure S20 DFT calculations. (a) Illustration of dementalization and (b) calculated dementalization energies

Figure S21 ORR polarization curves of CoIn-N-C, Co-N-C and In-N-C in O_2-satureted 0.1 mol L^{-1} $HClO_4$ before (solid lines) and after (dashed lines) poisoned by KSCN

Figure S22 Optimized structure of SCN^--adsorbed s-Co and d-CoInOH

Figure S23 Photograph of the flow cells. (a) three-electrode and (b) two-electrode three-phase flow cell setup

Figure S24 Calibration curve for UV-vis spectrophotometric determination of Ce^{4+} in aqueous solution. Note that a.u. represents arbitrary units.

Figure S25 Polarization curve for CoIn-N-C in three-electrode flow cell. The solution resistance (R_s) of 18.8±0.3 Ω was determined by electrochemical impedance spectroscopy and the error represents the mean and standard deviation error of R_s measurements. iR correction can be achieved by subtracting the iR value from the measured potentials at applied each current density

Figure S26 Analysis of polarization origin in two-electrode three-phase flow cells with In-N-C as cathode catalyst, where the resistance of 2.00±0.10 Ω was 100% compensated

Figure S27 Two-electrode flow cell performances. Faradaic efficiencies (FE) and H_2O_2 production rate (k_{H2O2}) evaluated in two-electrode flow cell operated under different current density. The error bar represents the standard error of two independent tests. The error bar represents the standard error of two independent tests

Table S1 Adsorption energies of OOH, OH and O on Co and Mp atoms from dual-atom d-CoMp models and adsorption energies of OOH on OH-blocked dual-atom d-CoMpOH models

Table S2 Co valance electron number (n_{ve}) in s-CoVac and d-CoMpOH models

Table S3 The adsorption energy of OOH (ΔG_{OOH}) and limiting potential (U_L), representing

the potential below which the 2e-ORR become an exothermic reaction, on single atom models (s-Co, s-CoVac), dual-atom models (d-CoAl, d-CoGa, d-CoIn) and OH-blocked dual-atom models (d-CoAlOH, d-CoGaOH, d-CoInOH)

Table S4 Metal contents in the catalysts detected by ICP-OES

Table S5 Fitting parameters of Co K-edge and In K-edge EXAFS of CoIn-N-C

Table S6 Comparison of the H_2O_2 production performance by RRDE test

Table S7 Adsorption energy of SCN (ΔG_{SCN}) and bond length ($d_{Co\text{-}S}$ or $d_{In\text{-}S}$)

Table S8 Comparison of the H_2O_2 production performance in flow cells

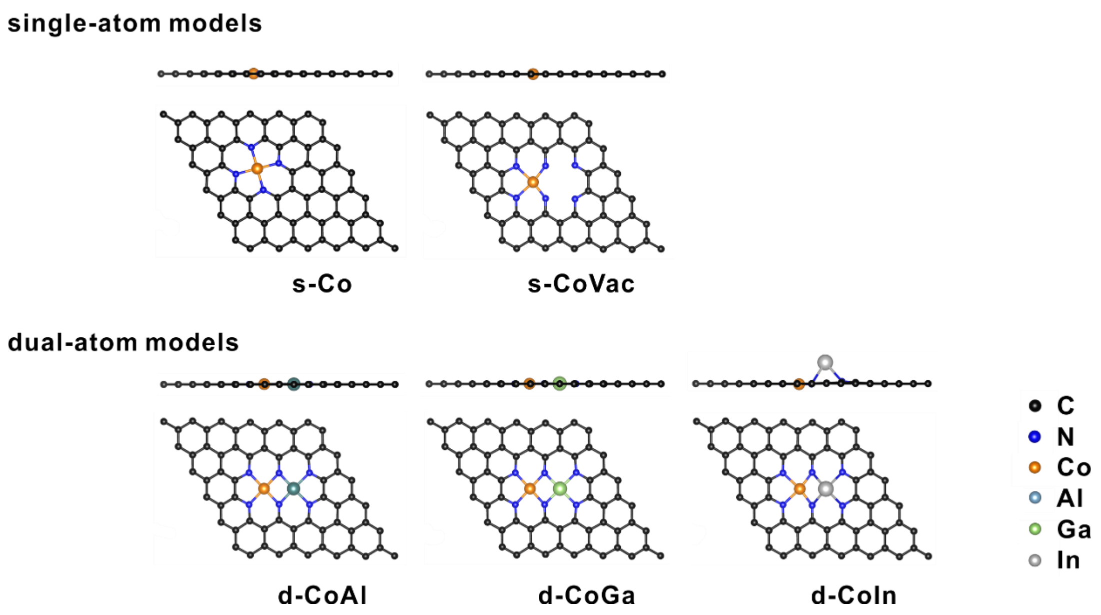

Figure S1 Optimized structure of the single atom models (s-Co, s-CoVac), and the dual-atom models (d-CoAl, d-CoGa, d-CoIn)

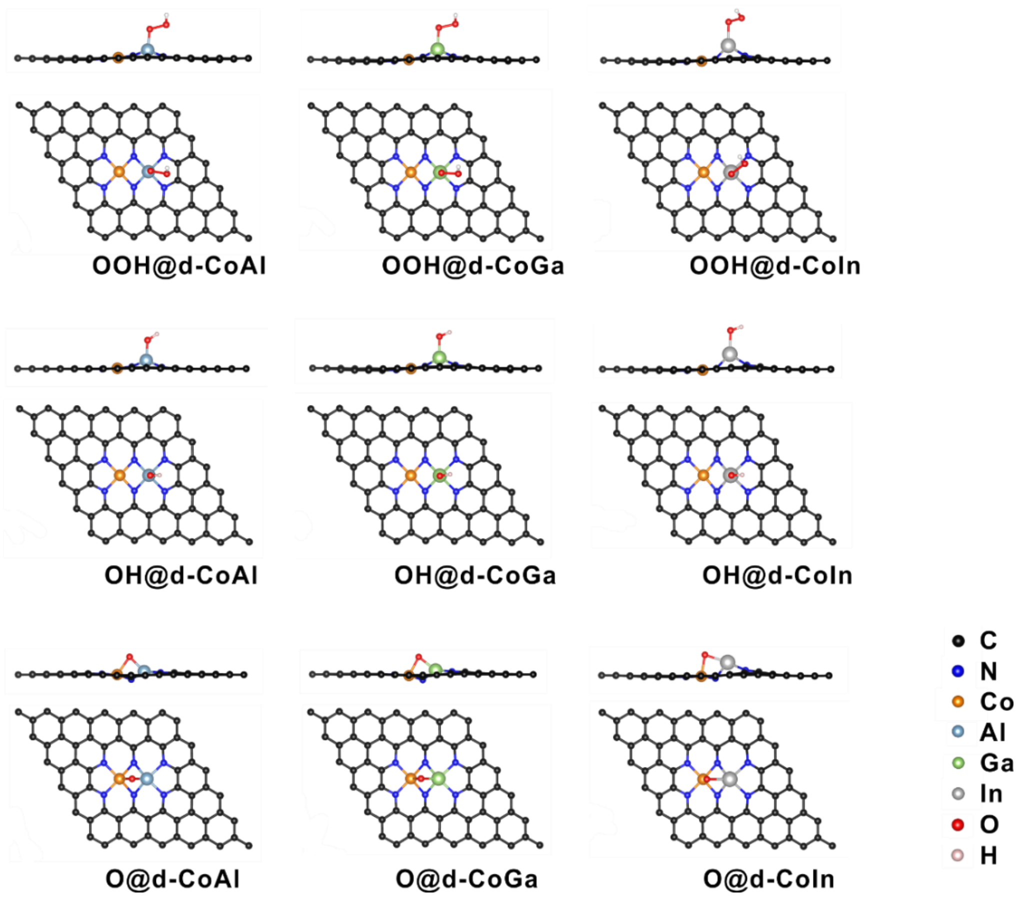

Figure S2 Optimized structure of OOH-, OH- and O-adsorbed dual-atom models (d-CoAl, d-CoGa, d-CoIn)

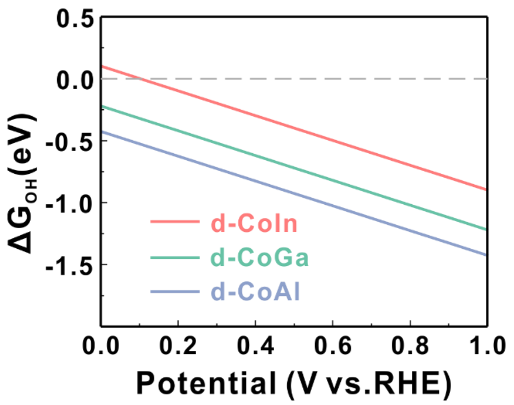

Figure S3 Adsorption energy of OH (ΔG_{OH}) on the three d-CoMp models as a function of potential

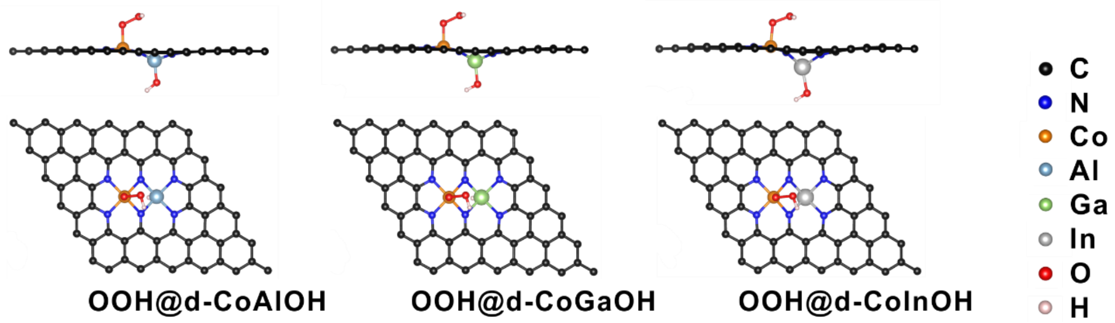

Figure S4 Optimized structure of OOH-adsorbed dual-atom models blocked by OH (d-CoAlOH, d-CoGaOH, d-CoInOH)

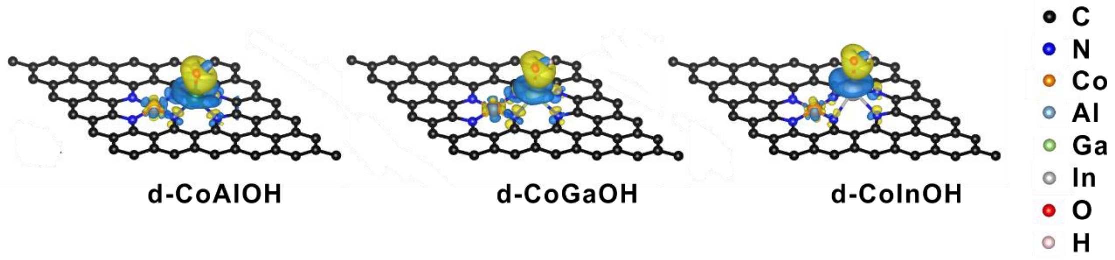

Figure S5 Charge density difference diagrams of d-CoMpOH, demonstrating the change of charge density upon introduction of MpOH moieties. The isosurface is set to be 0.003 e bohr^{-3}, where blue and yellow area represent charge depletion and accumulation, respectively.

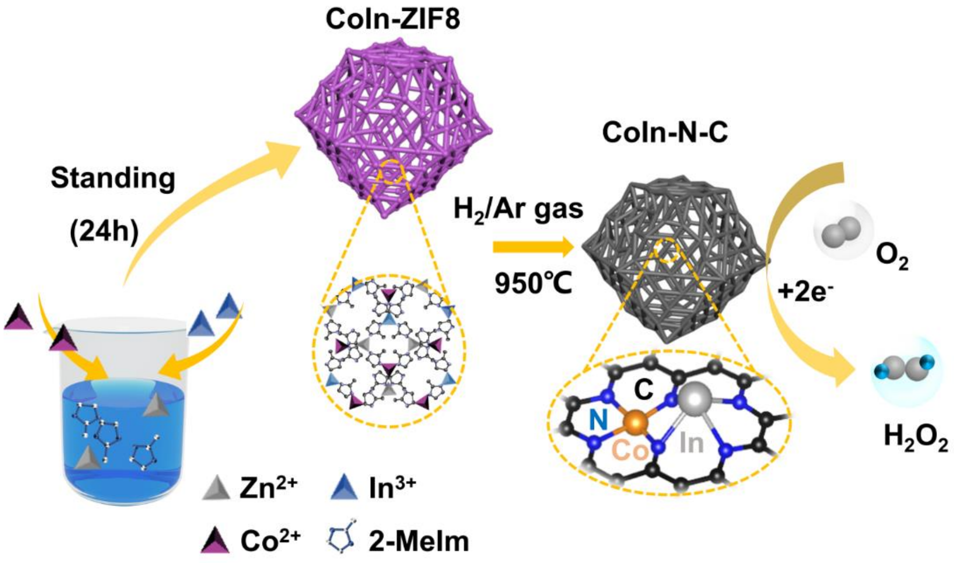

Figure S6 Synthesis process of CoIn-N-C

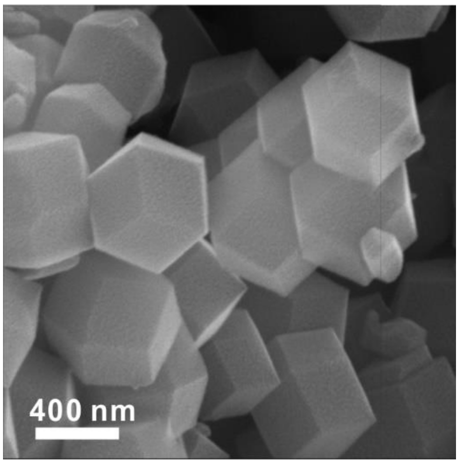

Figure S7 SEM image of the CoIn-ZIF8 precursor

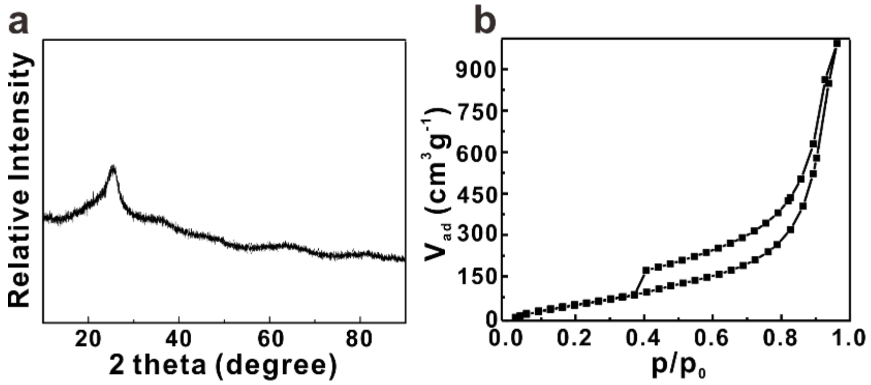

Figure S8 Structural characterization of CoIn-N-C (a) X-ray diffraction pattern, (b) N_2 adsorption−desorption isotherms of CoIn-N-C

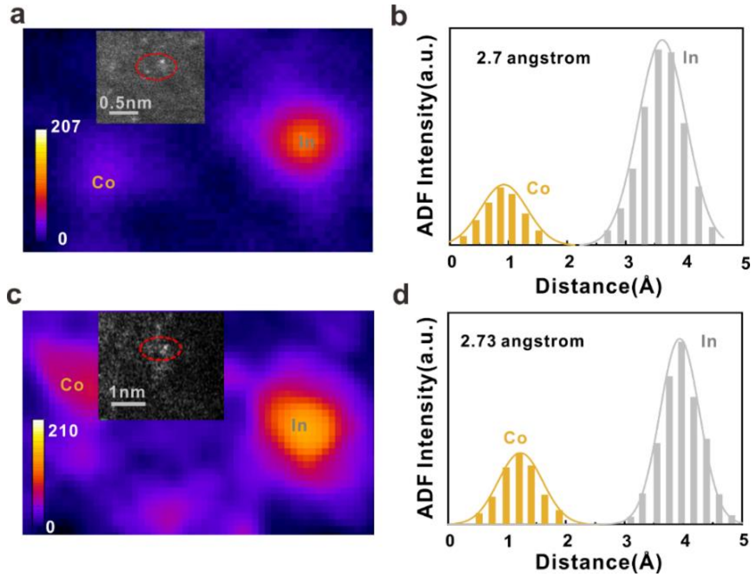

Figure S9 Colored raster graphic of other select regions in Figure 2d (a) (c) Z-contrast difference of the Co-In atomic pairs from CoIn-N-C. (b) (d) corresponding intensity profile Note that a.u. represents arbitrary units.

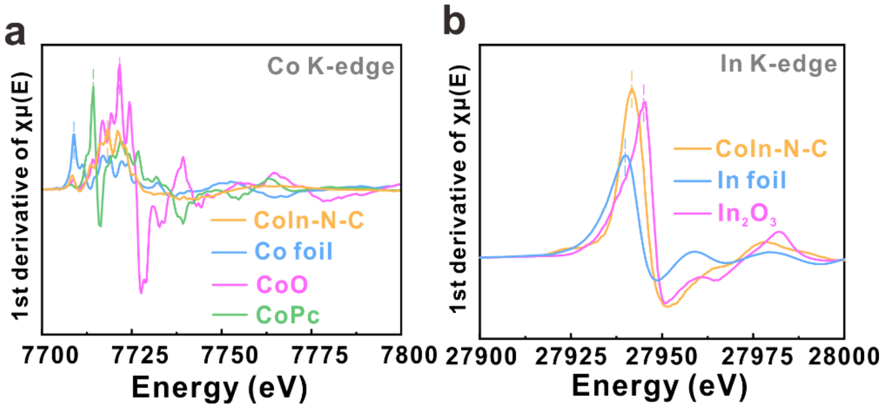

Figure S10 Co and In K-edge XANES analysts. (a) 1st derivatives of Co K-edge XANES for Co, CoO, CoPc, CoIn-N-C and (b) 1st derivatives of In K-edge XANES spectra for In, In_2O_3 and CoIn-N-C

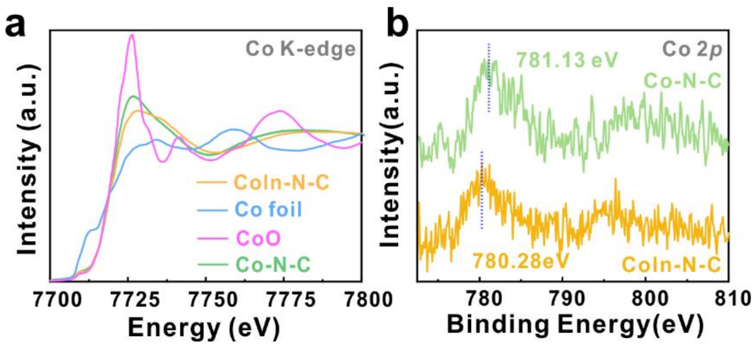

Figure S11 XANES and XPS spectras for the Co-NC. (a) Co K-edge XANES and (b) Co 2*p* XPS spectra of Co-N-C and CoIn-N-C. Note that a.u. represents arbitrary units.

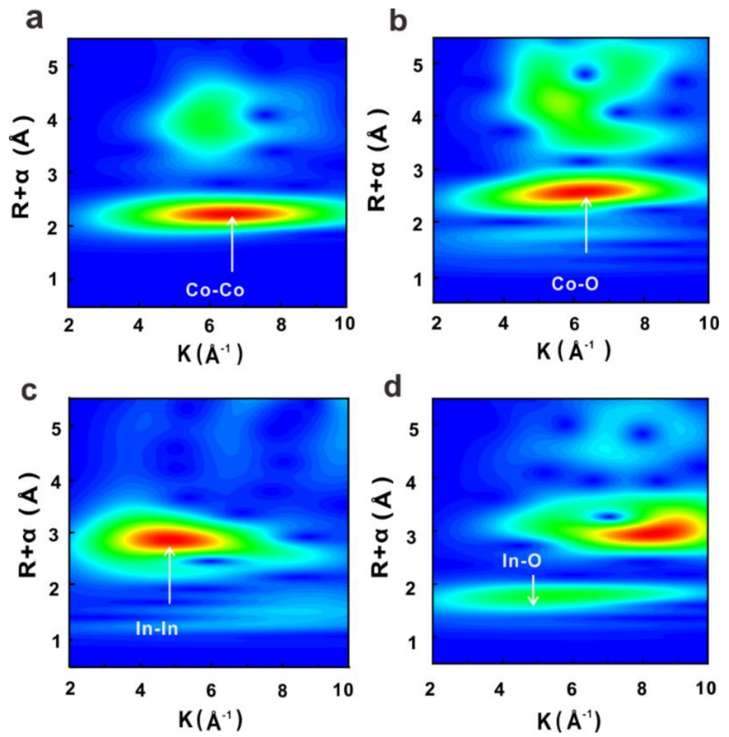

Figure S12 Wavelet transform (WT) contour plots of Co K edge EXAFS of (a) Co, (b) CoO, and In K edge EXAFS of (c) In, (d) In_2O_3

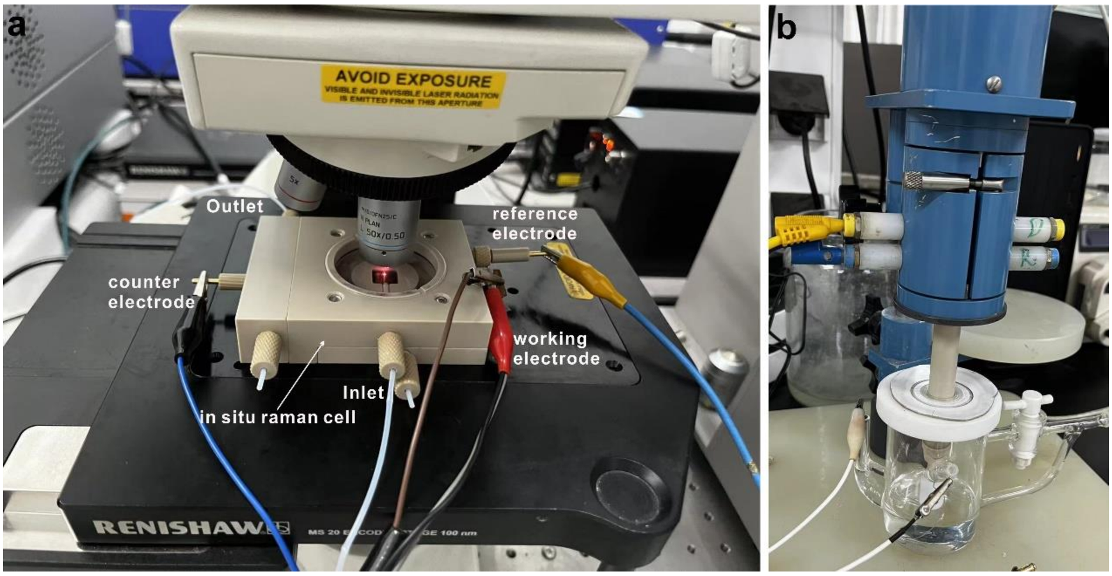

Figure S13 Photograph of in situ flow cell with optics window and electrochemical cell set-up. (a) three-electrode in situ Raman flow cell and (b) three-electrode electrochemical cell in working conditions

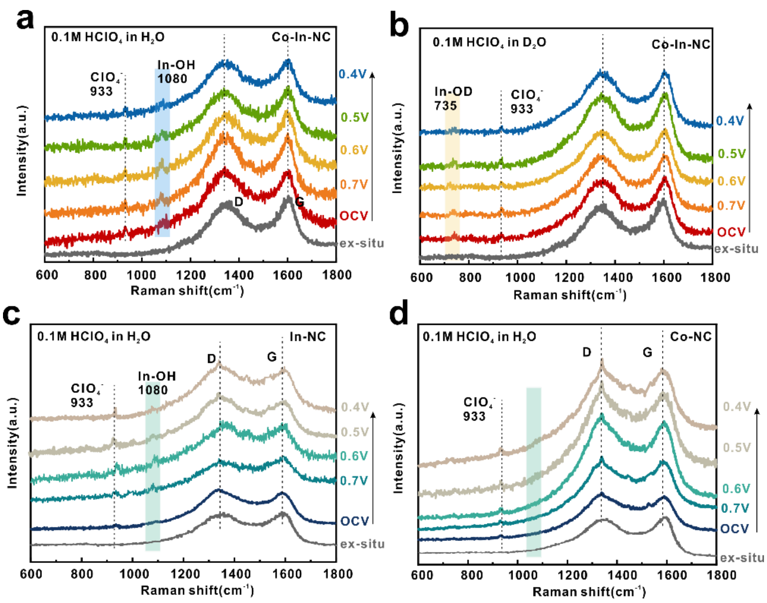

Figure S14 In situ EC-SHINERS results of CoIn-N-C Co-NC and In-NC at different condition. Raman spectra of (a) CoIn-N-C, (c) In-N-C and (d) Co-N-C recorded in ambient air (ex-situ) and Ar saturated 0.1 M $HClO_4$ aqueous solution and (b) CoIn-N-C in D_2O solution at different applied potential. Note that a.u. represents arbitrary units.

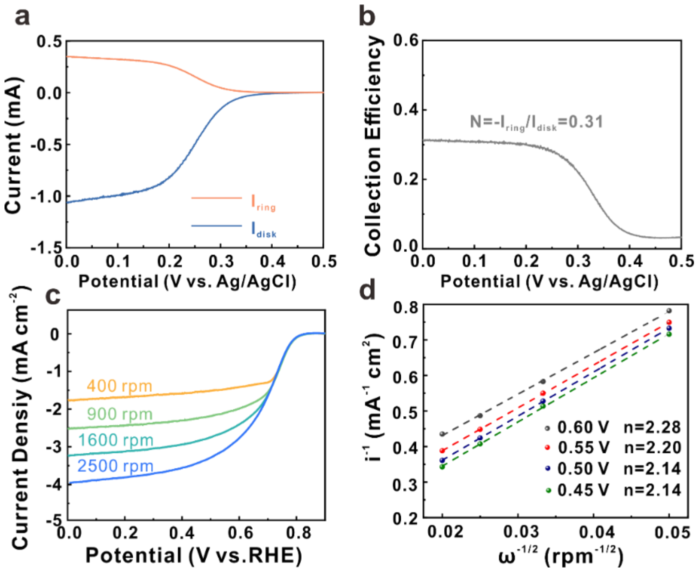

Figure S15 Electrochemical characterization of CoIn-N-C.(a) Polarizing curves recorded on CoIn-N-C coated disk electrode during potential scan and current response on the Pt ring electrode with a constant potential of 0.6 V in a mixture solution of 0.1 mol L^{-1} KCl and 10 mmol L^{-1} $K_3Fe(CN)_6$ at 1600 rpm, and (b) the experimentally determined collection efficiency (N), (c) ORR polarization curves of CoIn-N-C at different rotating rates in O_2-satureted 0.1 mol L^{-1} $HClO_4$, and (d) the corresponding K-L plots for the calculation of electron transfer number (n)

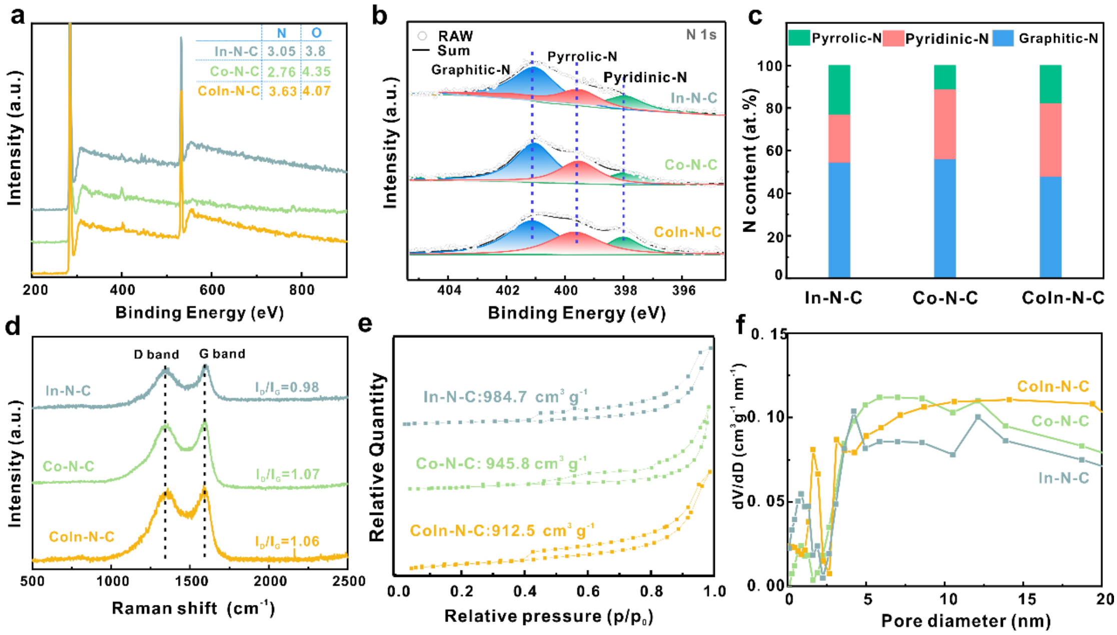

Figure S16 Physical characterization of CoIn-N-C Co-N-C and In-NC. (a) XPS survey spectra of Co-N-C, CoIn-N-C and In-NC (b) N 1*s* XPS spectra and fitting results. (c) N content ratio, (d) Raman spectra of Co-N-C, In-NC and CoIn-NC. (e) N_2 adsorption/desorption isotherms and (f) pore distribution curves. Note that a.u. represents arbitrary units.

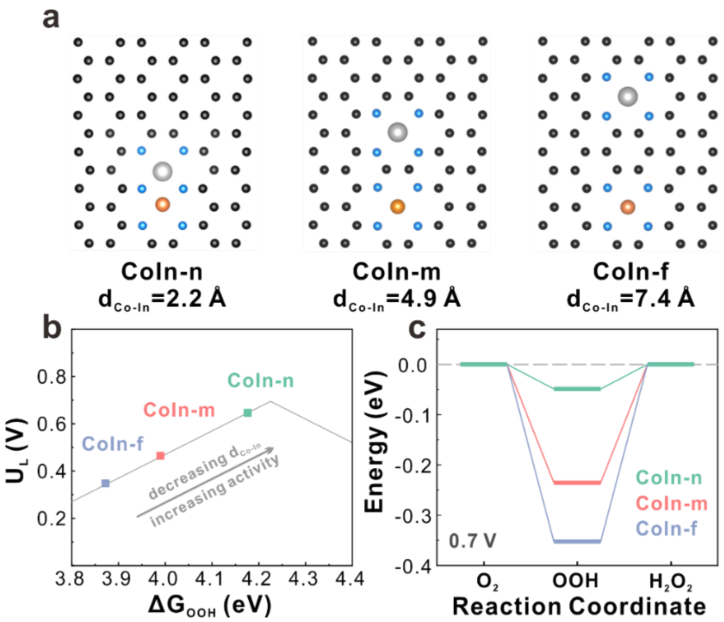

Figure S17 DFT calculations. (a) Dual atomic models with short (CoIn), middle (CoIn-m) and far (CoIn-f) distances and (b) calculated volcano plot and (c) free energy diagram.

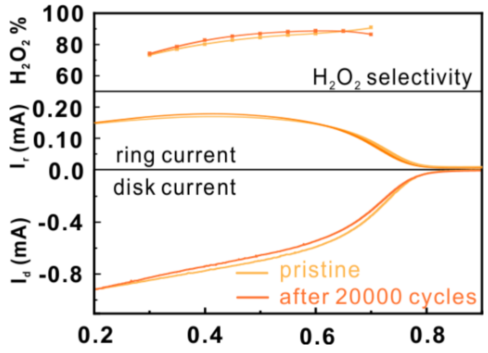

Figure S18 H_2O_2 production performance of CoIn-N-C before and after the accelerated degradation tests (ADTs)

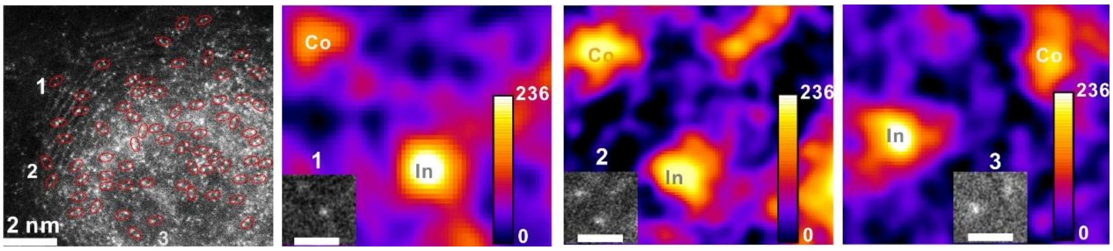

Figure S19 HAADF-STEM images and colored raster graphic of Co-In pair of catalysts after ADTS. The scale bars in the enlarge images (marked areas 1 2 3) are 0.35 nm.

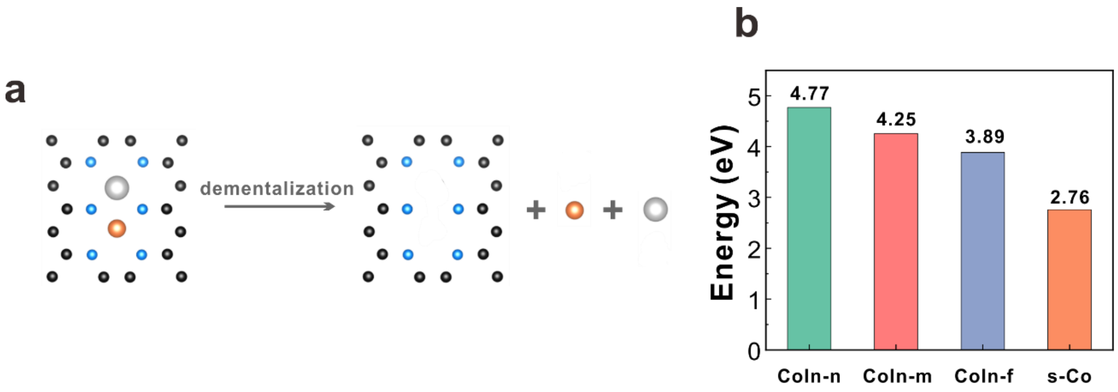

Figure S20 DFT calculations. (a) Illustration of dementalization and (b) calculated dementalization energies

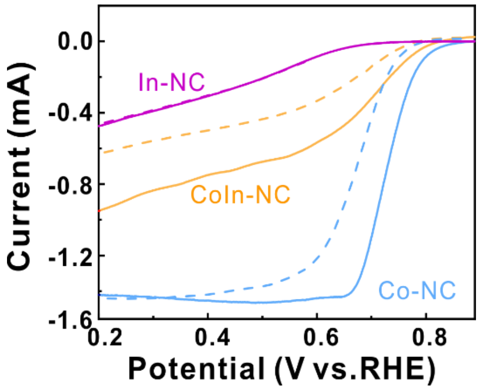

Figure S21 ORR polarization curves of CoIn-N-C, Co-N-C and In-N-C in O_2-satureted 0.1 mol L^{-1} $HClO_4$ before (solid lines) and after (dashed lines) poisoned by KSCN

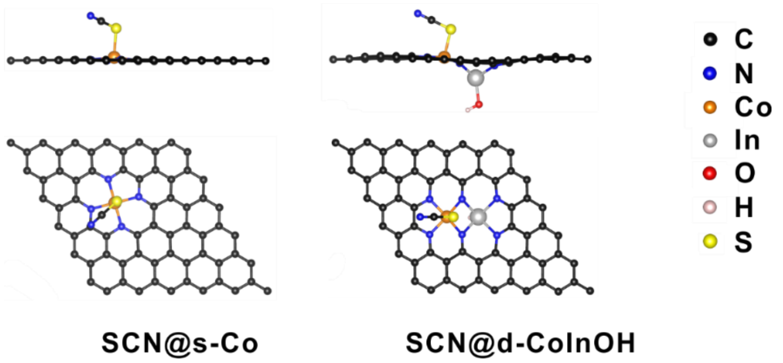

Figure S22 Optimized structure of SCN^--adsorbed s-Co and d-CoInOH

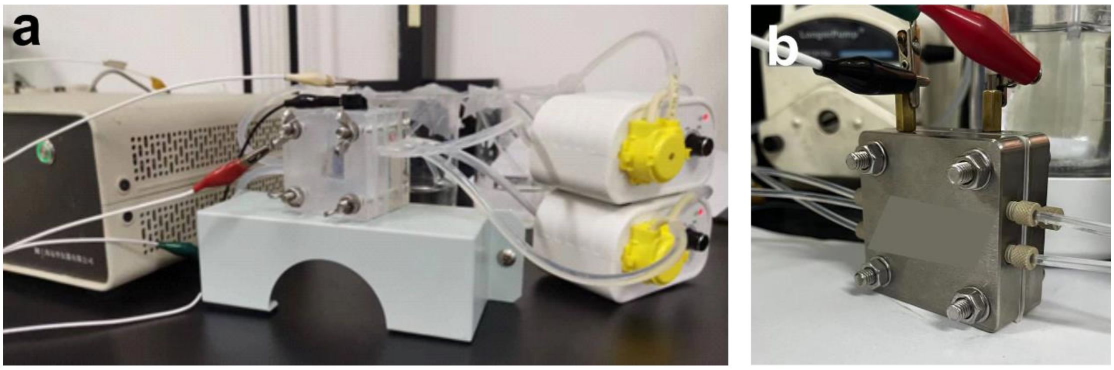

Figure S23 Photograph of the flow cells. (a) three-electrode and (b) two-electrode three-phase flow cell setup

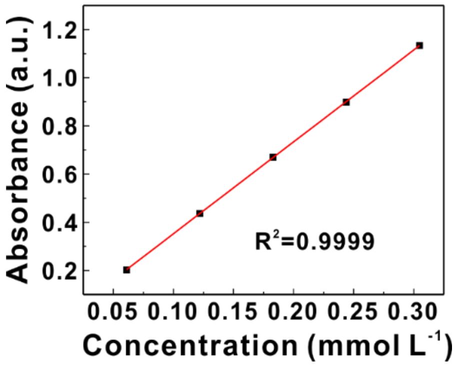

Figure S24 Calibration curve for UV-vis spectrophotometric determination of Ce^{4+} in aqueous solution. Note that a.u. represents arbitrary units

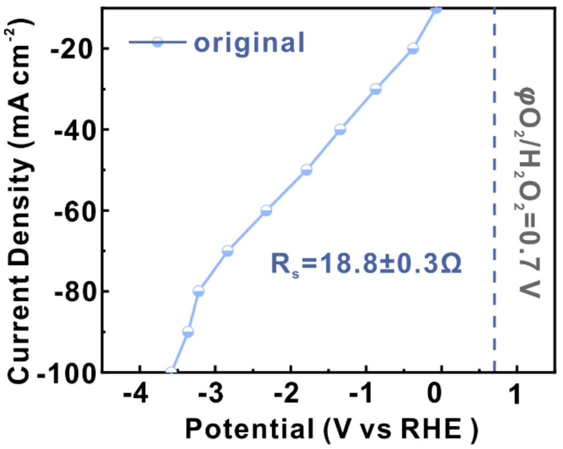

Figure S25 Polarization curve for CoIn-N-C in three-electrode flow cell. The solution resistance (R_s) of 18.8±0.3 Ω was determined by electrochemical impedance spectroscopy and the error represents the mean and standard deviation error of R_s measurements. iR correction can be achieved by subtracting the iR value from the measured potentials at applied each current density.

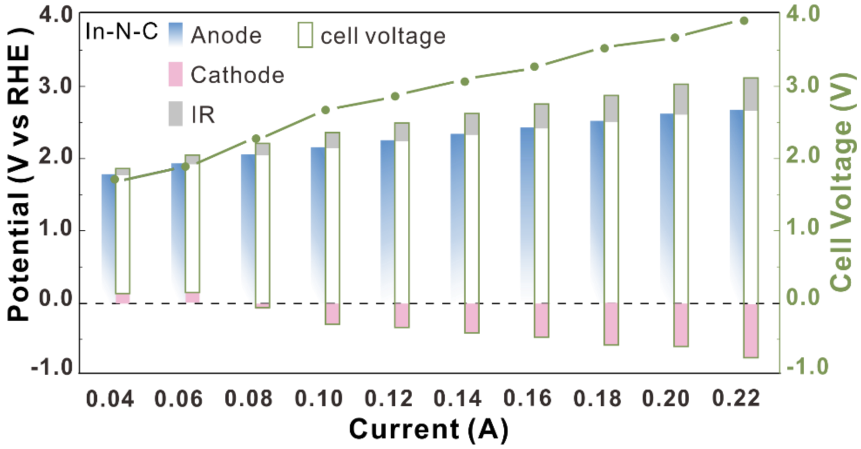

Figure S26 Analysis of polarization origin in two-electrode three-phase flow cells with In-N-C as cathode catalyst, where the resistance of 2.00±0.10 Ω was 100% compensated

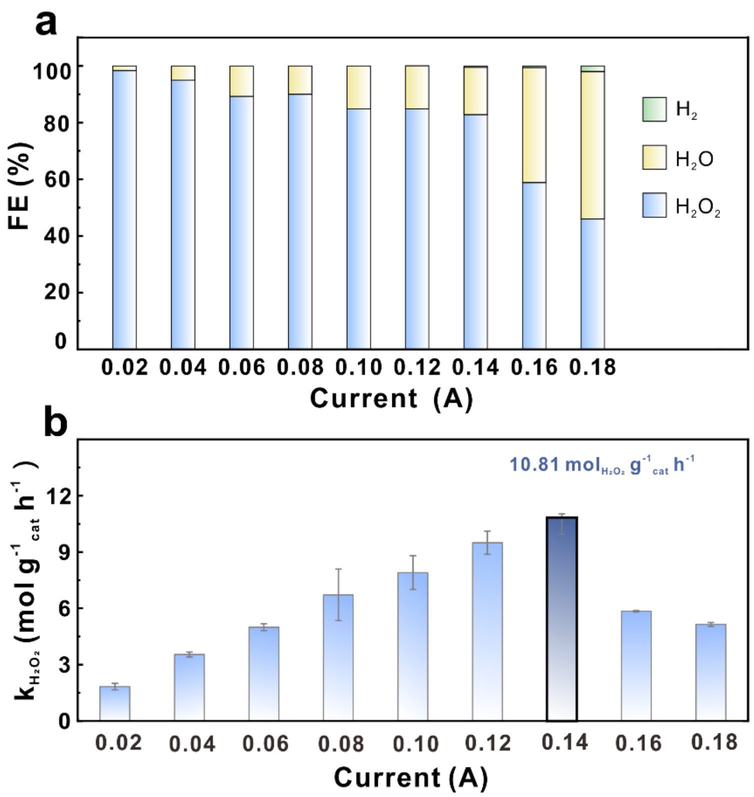

Figure S27 Two-electrode flow cell performances. Faradaic efficiencies (FE) and H_2O_2 production rate (k_{H2O2}) evaluated in two-electrode flow cell operated under different current density. The error bar represents the standard error of two independent tests

Table S1 Adsorption energies (ΔG_{ad}) of OOH, OH and O on Co and Mp atoms from dual-atom d-CoMp models and ΔG_{ad} of OOH on OH-blocked dual-atom d-CoMpOH models

	**ΔG_{ad} on d-CoAl (eV)**	**ΔG_{ad} on d-CoGa (eV)**	**ΔG_{ad} on d-CoIn (eV)**
**OOH**	3.938^{Co}<br>2.986^{Al}	3.907^{Co}<br>3.107^{Ga}	3.953^{Co}<br>3.373^{In}
**OH**	1.269^{Co}<br>-0.425^{Al}	1.096^{Co}<br>-0.220^{Ga}	1.062^{Co}<br>0.102^{In}
**O**	1.808^{b}	1.876^{b}	2.237^{b}
	**ΔG_{ad} on d-CoAlOH (eV)**	**ΔG_{ad} on d-CoGaOH (eV)**	**ΔG_{ad} on d-CoInOH (eV)**
**OOH**	4.082^{Co}<br>4.749^{Al}	4.090^{Co}<br>4.882^{Ga}	4.176^{Co}<br>4.947^{In}

The superscript for each ΔG_{ad} refer to the adsorption site, and b refers to bridge mode.

Table S2 Co valance electron number (n_{ve}) in s-CoVac and d-CoMpOH models

	**s-CoVac**	**d-CoAlOH**	**d-CoGaOH**	**d-CoInOH**
$\boldsymbol{n_{ve}}$	8.475	8.606	8.601	8.577

Table S3 The adsorption energy of OOH (ΔG_{OOH}) and limiting potential (U_L), representing the potential below which the 2e-ORR become an exothermic reaction, on single atom models (s-Co, s-CoVac), dual-atom models (d-CoAl, d-CoGa, d-CoIn) and OH-blocked dual-atom models (d-CoAlOH, d-CoGaOH, d-CoInOH)

	**ΔG_{OOH} (eV)**	**U_L (V)**
**s-Co**	3.95431	0.42431
**s-CoVac**	4.3881	0.5319
**d-CoAl**	2.9863	-0.5437
**d-CoGa**	3.1072	-0.4228
**d-CoIn**	3.3732	-0.1568
**d-CoAlOH**	4.0818	0.5518
**d-CoGaOH**	4.0904	0.5604
**d-CoInOH**	4.1762	0.6462

Table S4 Metal contents in the catalysts detected by ICP-OES

**Catalysts**	**Co wt.%**	**In wt.%**
**CoIn-N-C**	0.9	1.053
**Co-N-C**	1.78	-
**In-N-C**	-	1.925

Table S5 Fitting parameters of Co K-edge and In K-edge EXAFS of CoIn-N-C

<table>
<tr><th>Sample</th><th>path</th><th>CN</th><th>R (Å)</th><th>σ^2 (10^{-3} Å)</th><th>ΔE_0 (eV)</th><th>R-factor</th></tr>
<tr><td rowspan="3">In K-edge</td><td>$In-N_1$</td><td>2.00±0.03</td><td>2.30±0.05</td><td>7.0±1.6</td><td rowspan="3">1.0±0.7</td><td rowspan="3">0.003</td></tr>
<tr><td>$In-N_2$</td><td>2.00±0.12</td><td>2.50±0.06</td><td>7.7±1.3</td></tr>
<tr><td>In-Co</td><td>1.00±0.11</td><td>2.70±0.11</td><td>2.1±1.3</td></tr>
<tr><td rowspan="3">Co K-edge</td><td>$Co-N_1$</td><td>1.99±0.11</td><td>1.90±0.12</td><td>8.3±0.5</td><td rowspan="3">1.7±0.9</td><td rowspan="3">0.013</td></tr>
<tr><td>$Co-N_2$</td><td>2.00±0.15</td><td>2.00±0.19</td><td>9.5±1.6</td></tr>
<tr><td>Co-In</td><td>0.92±0.32</td><td>2.70±0.14</td><td>5.0±2.5</td></tr>
</table>

CN is the coordination number; *R* is the interatomic distance; σ^2 is the Debye-Waller factor; ΔE_0 is the energy shift; R-factor reflects the deviation error of the fitting

Table S6 Comparison of the H_2O_2 production performance by RRDE test

**Samples**	***i*@0.65 V (mA cm^{-2})**	**H_2O_2 %**	**i_{H2O2} @0.65 V (mA cm^{-2})**	**electrolyte**	**Ref.**
CoIn-N-C	2	96	1.920	0.1 M $HClO_4$	This work
CoN_4/VG	0.48	98	0.470	0.1 M $HClO_4$	1
Pd^+ OCNT	0.2	95	0.190	0.1 M $HClO_4$	2
h-Pt_1-CuS_x	0.1	97	0.097	0.1 M $HClO_4$	3
Co NOC	0	95	0.000	0.1 M $HClO_4$	4
CoS_2	0.9	80	0.720	0.05 M H_2SO_4	5
O-$CoSe_2$	1	80	0.800	0.05 M H_2SO_4	6
Co_{SA}-N-CNTs	0.24	98	0.235	0.5 M H_2SO_4	7
Co-N SAC_{Dp}	0.13	96	0.125	0.1 M $HClO_4$	8
Co-NC	0.3	95	0.285	0.1 M $HClO_4$	9
Co-N/HPC	1.5	95	1.425	0.1 M KOH	10
Co_1-NG(O)	2	80	1.600	0.1 M KOH	11
W_1/NO-C	0.9	95	0.855	0.1 M KOH	12
Pd_1-NC	1.75	96	1.680	0.1 M KOH	13
In SAs/NSBC	2.14	95	2.033	0.1 M KOH	14
Mo_1/OSG-H	2.35	95	2.233	0.1 M KOH	15
Co-POC-O	1.81	90	1.629	0.1 M KOH	16
Co_{NPs}@N/C	2.4	90	2.160	0.1 M KOH	17
FeS-$CFs_{7:3}$	1.6	90	1.440	0.1 M KOH	18
CQD	1.61	95	1.530	0.1 M KOH	19
Ni MOF NSs-6	1.21	98	1.186	0.1 M KOH	20
$Pb(NiWMnNbZrTi)_{1/6}O_3$	1	97	0.970	0.1 M KOH	21

Table S7 Adsorption energy of SCN^- (ΔG_{SCN}) and Co-S or In-S bond length ($d_{Co\text{-}S}$ or $d_{In\text{-}S}$)

	ΔG_{SCN} (eV)	$d_{Co\text{-}S}$ (Å)	$d_{In\text{-}S}$ (Å)
**s-Co**	-3.7425	2.25	/
**Co in d-CoInOH**	-3.5033	2.22	/
**In in d-CoInOH**	-2.5263	/	4.24

Table S8 Comparison of the H_2O_2 production performance in flow cells

Samples	k_{H2O2} (mol g^{-1} h^{-1})	FE_{H2O2} (%)	Electrolyte	Ref.
**CoIn-N-C**	9.68	80	0.1M $HClO_4$	This work
**CoN_4/VG**	4	95	0.1M $HClO_4$	1
**Co-N SAC_{Dp}**	0.7	84	0.1 M $HClO_4$	8
**BP/$CoSe_2$**	0.8	90	0.1 M $HClO_4$	22
**Co_{SA}-N-CNTs**	0.9	90	0.5 M H_2SO_4	7
**CB-10%**	3.66	90	solid electrolyte	23
**NADE**	0.23	67	0.05 M Na_2SO_4	24
**Co-N-C**	3.4	96	0.5 M NaCl	25
**Co-N-C**	4.3	50	0.1M KOH	26
**In SAs/NSBC**	6.72	80	0.1M KOH	14
**Ni-N_2O_2/C**	5.9	91	0.1M KOH	27
**NPC950/GDE**	7.7	80	0.1M KOH	28
**N-FLG-8**	9.66	100	0.1M KOH	29
**NiB2**	4.75	93	0.1M KOH	30
**CMK3**	2.47	95	0.1M KOH	31
**CoN_4-PC**	11.2	92	0.1M KOH	32

Supplementary References

1. Lin, Z. *et al.* Atomic Co decorated free-standing graphene electrode assembly for efficient hydrogen peroxide production in acid. *Energy Environ. Sci.* **15**, 1172–1182 (2022).
2. Chang, Q. *et al.* Promoting H_2O_2 production via 2-electron oxygen reduction by coordinating partially oxidized Pd with defect carbon. *Nat. Commun.* **11**, 2178 (2020).
3. Shen, R. *et al.* High-Concentration Single Atomic Pt Sites on Hollow CuSx for Selective O2 Reduction to H_2O_2 in Acid Solution. *Chem* **5**, 2099–2110 (2019).
4. Tang, C. *et al.* Tailoring Acidic Oxygen Reduction Selectivity on Single-Atom Catalysts via Modification of First and Second Coordination Spheres. *J. Am. Chem. Soc.* **143**, 7819–7827 (2021).
5. Sheng, H. *et al.* Electrocatalytic Production of H_2O_2 by Selective Oxygen Reduction Using Earth-Abundant Cobalt Pyrite (CoS_2). *ACS Catal.* **9**, 8433–8442 (2019).
6. Sheng, H. *et al.* Stable and selective electrosynthesis of hydrogen peroxide and the electro-Fenton process on $CoSe_2$ polymorph catalysts. *Energy Environ. Sci.* **13**, 4189–4203 (2020).
7. Liu, W. *et al.* Tuning the atomic configuration of Co-N-C electrocatalyst enables highly-selective H2O2 production in acidic media. *Appl. Catal. B Environ.* **310**, 121312 (2022).
8. Chen, S. *et al.* Identification of the Highly Active Co–N_4 Coordination Motif for Selective Oxygen Reduction to Hydrogen Peroxide. *J. Am. Chem. Soc.* **144**, 14505–14516 (2022).
9. Gao, J. *et al.* Enabling Direct H_2O_2 Production in Acidic Media through Rational Design

of Transition Metal Single Atom Catalyst. *Chem* **6**, 658–674 (2020).

10. Tian, Y. *et al.* Edge‐hosted Atomic Co−N_4 Sites on Hierarchical Porous Carbon for Highly Selective Two‐electron Oxygen Reduction Reaction. *Angew. Chem. Int. Ed.* **61**, e202213296 (2022).
11. Jung, E. *et al.* Atomic-level tuning of Co–N–C catalyst for high-performance electrochemical H_2O_2 production. *Nat. Mater.* **19**, 436–442 (2020).
12. Zhang, F. *et al.* High-Efficiency Electrosynthesis of Hydrogen Peroxide from Oxygen Reduction Enabled by a Tungsten Single Atom Catalyst with Unique Terdentate N_1O_2 Coordination. *Adv. Funct. Mater.* **32**, 2110224 (2022).
13. Wang, N. *et al.* Highly Selective Oxygen Reduction to Hydrogen Peroxide on a Carbon-Supported Single-Atom Pd Electrocatalyst. *ACS Catal.* **12**, 4156–4164 (2022).
14. Zhang, E. *et al.* Engineering the Local Atomic Environments of Indium Single‐Atom Catalysts for Efficient Electrochemical Production of Hydrogen Peroxide. *Angew. Chem. Int. Ed.* **61**, e202117347 (2022).
15. Tang, C. *et al.* Coordination Tunes Selectivity: Two‐Electron Oxygen Reduction on High-Loading Molybdenum Single‐Atom Catalysts. *Angew. Chem. Int. Ed.* **59**, 9171–9176 (2020).
16. Li, B., Zhao, C., Liu, J. & Zhang, Q. Electrosynthesis of Hydrogen Peroxide Synergistically Catalyzed by Atomic Co-N_x-C Sites and Oxygen Functional Groups in Noble-Metal-Free Electrocatalysts. *Adv. Mater.* **31**, 1808173 (2019).
17. Wu, J. *et al.* Highly Selective O_2 Reduction to H_2O_2 Catalyzed by Cobalt Nanoparticles Supported on Nitrogen-Doped Carbon in Alkaline Solution. *ACS Catal.* **11**, 5035–5046

(2021).

18. Xiang, F. *et al.* Enhanced Selectivity in the Electroproduction of H_2O_2 via F/S Dual-Doping in Metal-Free Nanofibers. *Adv. Mater.* 2208533 (2022) doi:10.1002/adma.202208533.

19. Guo, Y. *et al.* Ultrahigh oxygen-doped carbon quantum dots for highly efficient H_2O_2 production *via* two-electron electrochemical oxygen reduction. *Energy Environ. Sci.* **15**, 4167–4174 (2022).

20. Wang, M. *et al.* An Efficient Interfacial Synthesis of Two-Dimensional Metal–Organic Framework Nanosheets for Electrochemical Hydrogen Peroxide Production. *Angew. Chem. Int. Ed.* **60**, 11190–11195 (2021).

21. Chen, Z. *et al.* Entropy Enhanced Perovskite Oxide Ceramic for Efficient Electrochemical Reduction of Oxygen to Hydrogen Peroxide. *Angew. Chem. Int. Ed.* **61**, e202200086 (2022).

22. Zheng, Y.-R. *et al.* Black Phosphorous Mediates Surface Charge Redistribution of $CoSe_2$ for Electrochemical H_2O_2 Production in Acidic Electrolytes. *Adv. Mater.* **34**, 2205414 (2022).

23. Xia, C., Xia, Y., Zhu, P., Fan, L. & Wang, H. Direct electrosynthesis of pure aqueous H_2O_2 solutions up to 20% by weight using a solid electrolyte. *Science* **366**, 226–231 (2019).

24. Zhang, Q. *et al.* Highly efficient electrosynthesis of hydrogen peroxide on a superhydrophobic three-phase interface by natural air diffusion. *Nat. Commun.* **11**, 1731 (2020).

25. Zhao, Q. *et al.* Approaching a high-rate and sustainable production of hydrogen peroxide: oxygen reduction on Co-N-C single-atom electrocatalysts in simulated seawater. *Energy Environ. Sci.* **14**, 5444–5456 (2021).

26. Sun, Y. *et al.* Activity–Selectivity Trends in the Electrochemical Production of Hydrogen Peroxide over Single-Site Metal-Nitrogen-Carbon Catalysts. *J. Am. Chem. Soc.* **141**, 12372–12381 (2019).

27. Wang, Y. *et al.* High - Efficiency Oxygen Reduction to Hydrogen Peroxide Catalyzed by Nickel Single-Atom Catalysts with Tetradentate N_2O_2 Coordination in a Three-Phase Flow Cell. *Angew. Chem. Int. Ed.* **59**, 13057–13062 (2020).

28. Cao, P. *et al.* Durable and Selective Electrochemical H_2O_2 Synthesis under a Large Current Enabled by the Cathode with Highly Hydrophobic Three-Phase Architecture. *ACS Catal.* **11**, 13797–13808 (2021).

29. Li, L. *et al.* Tailoring Selectivity of Electrochemical Hydrogen Peroxide Generation by Tunable Pyrrolic-Nitrogen-Carbon. *Adv. Energy Mater.* **10**, 2000789 (2020).

30. Wu, J. *et al.* Composition Engineering of Amorphous Nickel Boride Nanoarchitectures Enabling Highly Efficient Electrosynthesis of Hydrogen Peroxide. *Adv. Mater.* **34**, 2202995 (2022).

31. Wang, Y.-L. *et al.* One minute from pristine carbon to an electrocatalyst for hydrogen peroxide production. *J. Mater. Chem. A* **7**, 21329–21337 (2019).

32. Liu, J. *et al.* Single-atom CoN_4 sites with elongated bonding induced by phosphorus doping for efficient H_2O_2 electrosynthesis. *Appl. Catal. B Environ.* **324**, 122267 (2023).
